# Implementation of a high-throughput microfluidic platform for antimicrobial resistance surveillance in swine production systems

**DOI:** 10.1099/mgen.0.001755

**Published:** 2026-06-15

**Authors:** Judith Guitart-Matas, Yuliaxis Ramayo-Caldas, Olga González-Rodríguez, Noemí Giler-Baquerizo, Lourdes Migura-Garcia, Maria Ballester

**Affiliations:** 1 Joint Research Unit IRTA-UAB in Animal Health, Animal Health Research Centre (CReSA), Autonomous University of Barcelona (UAB), Bellaterra, 08193, Spain; 2 Institute of Agrifood Research and Technology (IRTA), Animal Health Program (CReSA), WOAH Collaborating Centre for the Research and Control of Emerging and Re-Emerging Swine Diseases in Europe, Autonomous University of Barcelona (UAB), Bellaterra, 08193, Spain; 3 Institute of Agrifood Research and Technology (IRTA), Animal Breeding and Genetics Program, Caldes de Montbui, 08140, Spain

**Keywords:** antimicrobial resistance, high-throughput, microfluidics, resistome, sequencing

## Abstract

Antimicrobial resistance poses a serious threat to public health worldwide and demands interventions with a One Health perspective. A key challenge is determining the collection of antimicrobial resistance genes of a specific environment, also known as the resistome. Surveillance and monitoring of the resistome are essential for tracking the emergence and dissemination of resistance mechanisms. In this study, we took advantage of shotgun metagenomics and metatranscriptomics sequencing data of piglets treated with different post-weaning diarrhoea treatments to generate an antimicrobial resistance gene catalogue of the pig gut microbiome during pre-weaning and post-weaning stages. The selected catalogue, comprising a total of 102 genes and representing the majority of antibiotic classes, has been implemented in the microfluidic Biomark^™^ X9 System and validated using total DNA and RNA extracted from piglets’ faecal samples. Additionally, this platform has been verified by demonstrating a strong and statistically significant correlation with resistome quantification data from both metagenomic and metatranscriptomic sequencing. Overall, the microfluidic qPCR platform implemented here demonstrated enhanced detection of low-abundance targets, successfully identifying genes and transcripts that remained below the stochastic detection threshold of shotgun sequencing. This approach enables high-throughput monitoring and surveillance of antimicrobial resistance, providing a critical tool to support the reduction of antimicrobial use in farms.

**Impact Statement**

The growing global health crisis of antimicrobial resistance demands urgent and coordinated actions across human, animal and environmental health domains. This study addresses this challenge by generating a comprehensive catalogue of 102 antimicrobial resistance genes from the pig gut microbiome. Implemented within a microfluidic qPCR platform, the catalogue enables rapid, sensitive and specific detection of resistance genes across most antibiotic classes. The Biomark^™^ X9 System demonstrated strong concordance with sequencing-based resistome quantification, confirming its technical accuracy and reliability within our analytical pipeline. Moreover, its enhanced specificity and sensitivity offer a practical alternative to traditional sequencing, facilitating rapid and efficient surveillance of antimicrobial resistance in agricultural settings. This advancement holds significant promise for improving antimicrobial resistance surveillance across livestock systems, contributing to global efforts to safeguard public health. The validated microfluidic qPCR platform represents a scalable and cost-effective solution for integrating resistome monitoring into routine farm management and policy frameworks to make informed decisions that would reduce unnecessary antimicrobial use, ultimately curbing the spread of resistance.

## Data Summary

Metagenomics and metatranscriptomics raw data have been submitted to the National Center for Biotechnology Information Sequence Read Archive with the BioProject accession number PRJNA1010706. A complete list of all specific accession numbers is provided in Table S1. All scripts are deposited in the following GitHub repository: https://github.com/judithguitart/Multi-omics-analysis.

## Introduction

The resistome, defined as the collective repertoire of genes and genetic elements associated with antimicrobial resistance (AMR), represents the genetic potential of a micro-organism or a community of micro-organisms to resist the effects of antimicrobial agents [1,2]. The emergence of AMR represents a serious public health threat at a global scale. Hence, understanding the resistome across diverse environments is fundamental for monitoring resistance mechanisms and developing effective strategies to mitigate the spread of AMR and its negative impact. For many years, standard methods for resistome surveillance have relied on culture-based techniques and phenotypic determination under laboratory conditions. However, the continuous development and enhanced availability of high-throughput molecular- and sequencing-based techniques offer the possibility of conducting a more comprehensive study of the resistome within a microbiome [3]. These technologies have enabled the identification of antimicrobial resistance genes (ARGs) across a wide range of environments, underscoring the need to tackle AMR through coordinated actions with a One Health perspective [4,6]. Within this paradigm, the livestock sector plays a critical role in the sustained antibiotic pressure over the years and its interconnectedness with human and environmental systems. Besides, the presence of ARGs in livestock poses a major concern due to their potential spread through the food chain and the consequential risks to public health [7].

In the swine industry, the post-weaning period is a critical phase for piglet survival and growth efficiency. At this stage, piglets are particularly vulnerable to infections due to the physiological, dietary and environmental changes that occur during this transition, leading to increased antimicrobial use [8,10]. In recent years, growing scientific interest in the gut microbiome has highlighted the detrimental effects of antimicrobials, disrupting microbial composition and negatively impacting animal health, including digestion and immunity functions [11,12]. Altogether, the need to develop strategies to reduce the use of antimicrobials in swine farms is inevitable. These strategies require integrated approaches, including improvements in biosecurity and husbandry practices, disease control measures, diagnostics and awareness, while ensuring animal health and welfare, and preserve productivity [13,14].

Previous research from our group studied the longitudinal and cross-sectional effects of different treatments commonly prescribed for post-weaning diarrhoea (PWD) on the microbiome and the resistome of pigs [15,16]. The study included seven different treatments: four antimicrobials (gentamicin, colistin, amoxicillin and trimethoprim/sulfamethoxazole), an oral attenuated vaccine, a control with the addition of acidifiers in the drinking water and an untreated control. The influence of these treatments on the microbiome and the resistome was analysed through shotgun metagenomics and shotgun metatranscriptomics sequencing at four sampling times (before weaning and 2 days, 2 weeks and 4 weeks post-treatment), as previously described [15]. In this study, resistome analyses from both metagenomic and metatranscriptomic datasets were compiled to generate an ARG catalogue from the pig gut microbiome. This catalogue has been implemented in a microfluidic qPCR platform, the Biomark^™^ X9 System, to detect ARGs and quantify their relative mRNA expression in pig faecal samples. The platform has been optimized to detect ARGs using total DNA and RNA extracted from faecal samples. A total of 96 assays, covering more than 100 ARGs, have been designed, validated and compared with available metagenomics and metatranscriptomics resistome data to estimate the potential of this tool to enhance AMR surveillance in farms.

## Methods

### Experimental design

Faecal samples were collected as described in Guitart-Matas *et al*., from an experimental setting, including a total of 210 piglets [15]. The farm of origin, located in Catalonia (Spain), was selected for previous records of PWD outbreaks. After weaning, piglets were divided into seven treatment groups, and one of them remained at the farm of origin (GG), which implemented a routine programme of amoxicillin treatment at the time of study. The remaining piglets were transferred to an experimental farm from the *Institut de Recerca i Tecnologia Agroalimentàries* (IRTA) that was previously decontaminated, cleaned and disinfected. Piglets were divided into six different treatment groups: trimethoprim/sulfamethoxazole (G1), colistin (G2), commercial oral *Escherichia coli* vaccine (G3), gentamicin (G4), untreated control with water acidification (G5) and untreated control (G6). Antibiotic treatments were selected based on previous epidemiological data from the farm and were applied orally in water for 5 days when individual animals from different groups showed clinical signs of mild diarrhoea. The signs started 11 days after arrival at the experimental farm. Dosages and concentrations of the cited antimicrobials were determined by the summary of product characteristics (SmPC). The commercial lyophilized vaccine (Coliprotec F4/F18, Elanco GmbH) included non-attenuated and non-pathogenic *E. coli* O8:K87 and O141:K94. It was applied orally in a single dose on the day of arrival at the experimental farm. For the acidification of the drinking water, phosphoric acid 75% was used (Serbonet Reductor pH, Inserbo, S.L.) from the arrival of the animals at the experimental farm until the end of the study. The same feed was supplied at the farm of origin and at the IRTA experimental farm. Faecal samples were collected from individual piglets on four different occasions: at the farm of origin 1 day before weaning and departure to the experimental farm (ST1), 3 days post-treatment (ST2), 2 weeks post-treatment (ST3) and 4 weeks post-treatment (ST4).

**Fig. 1.**
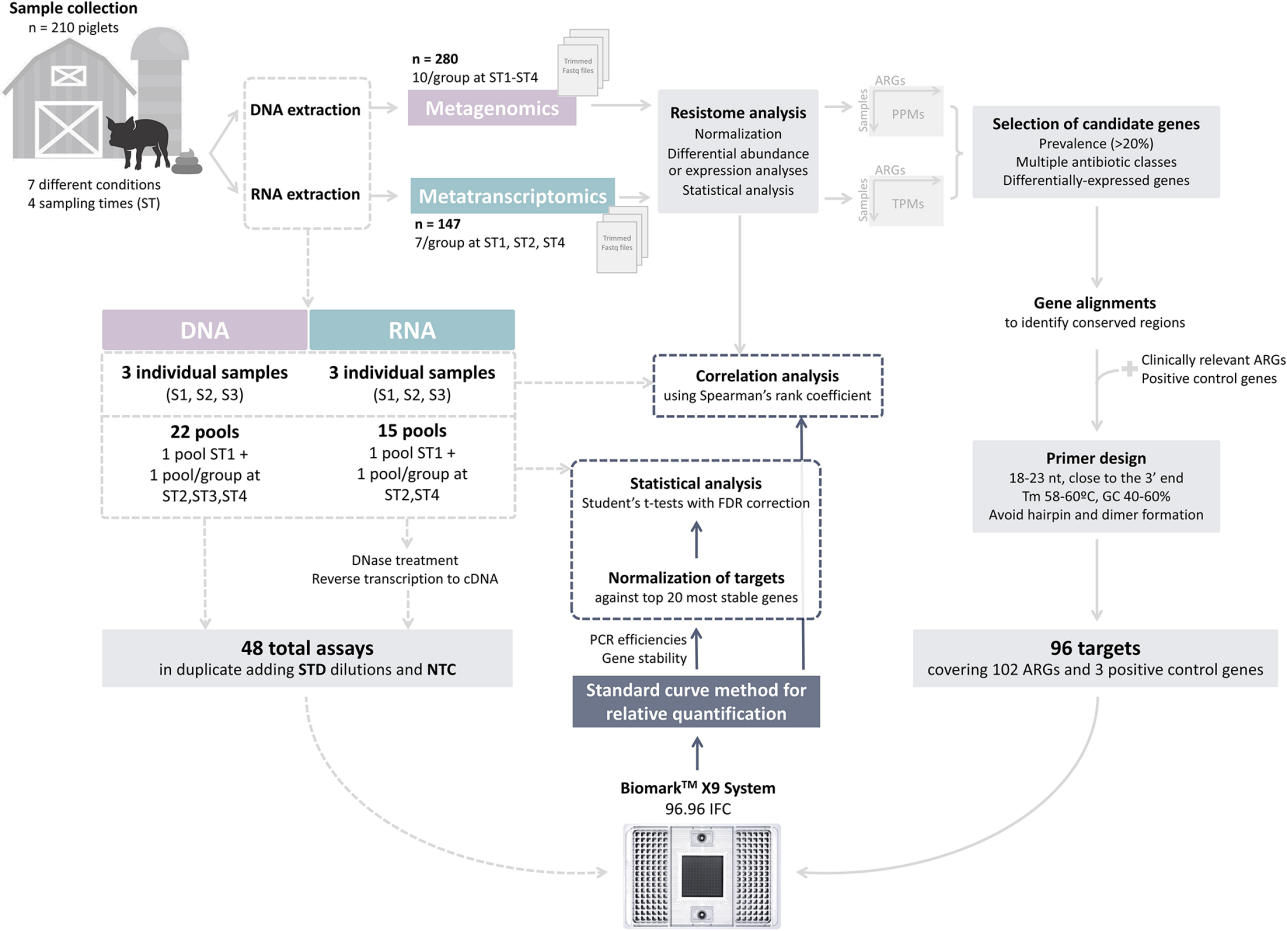
Overview of the methodological workflow. The diagram illustrates the experimental pipeline from sample collection to the implementation of the 96 target genes. The workflow also details the 48 assays assessed in the high-throughput microfluidic Biomark^™^ X9 System, including the downstream quantification analysis. ST, sampling time; STD, standard; NTC, no-template control; ARGs, antimicrobial resistance genes. ST1, 1 day before weaning; ST2, 3 days post-treatment; ST3, 2 weeks post-treatment; ST4, 4 weeks post-treatment.

The integrated workflow encompassing these samples and the subsequent molecular, bioinformatic and statistical analysis for the detection and quantification of specific ARGs using the Biomark^™^ X9 System is visually summarized in Fig. 1.

### Shotgun metagenomics and metatranscriptomics sequencing and resistome analyses

Total DNA and RNA from faecal samples were extracted and paired-end sequenced as previously described [15,16]. Specifically, shotgun metagenomics was performed on samples from ten animals per group at all sampling times (*n*=280), while shotgun metatranscriptomics was performed on seven animals per treatment group across three sampling times (*n*=147). Resistance profiles from metagenomics and metatranscriptomics data were determined with ResFinder v4.2.5 from quality-trimmed metagenomic and metatranscriptomic reads. Normalization and quantification were performed as described in Guitart-Matas *et al*. in p.p.m. [15] and transcripts per million [16].

### Selection of ARG candidates and primer design

For the generation of the ARG catalogue, selection of candidate genes was first based on the gene prevalence identified from the metagenomics data [15]. ARGs found in at least 20% of the samples were selected, and their individual expression patterns were also studied. Some ARGs with a prevalence lower than 20% were also selected to include genes conferring resistance to multiple antibiotic classes. Besides, ARGs identified directly from the metagenome-assembled genomes (MAGs) and differentially expressed genes associated with resistance identified with DESeq2 in Guitart-Matas *et al*. were also incorporated [16]. The ARG catalogue also included some clinically relevant ARGs (*bla*_CTX-M-1_, *bla*_CTX-M-9_, *bla*_CTX-M-12_, *bla*_CTX-M-14_, *bla*_CTX-M-15_, *bla*_CMY-2_, *bla*_OXA-48_, *bla*_OXA-181_, *bla*_NDM_, *bla*_IMP_, *bla*_VIM_, *bla*_GES_, *mecC*, *mcr-4*, *cfrA*, *poxtA*, *vanB*, *intl1-*LC1, *qepA*, *qnrA*, *qnrS2*, *qnrS5* and *qnrS6*) and three positive control genes for bacteria: the hypervariable region V7 of the 16S rRNA and the *gyrA* and *rpoD* genes.

As some ARGs have multiple accession gene IDs or variants, specific ResFinder accessions identified within our dataset were retrieved to perform gene alignments. Concerning selected ARGs not identified with ResFinder, such as the clinically relevant ARGs, all entries for each gene were downloaded from the Comprehensive Antibiotic Resistance Database (CARD). Gene alignments were performed with the Unipro UGENE alignment tool using the multiple sequence comparison by log-expectation (muscle) method to identify conserved regions to design primers [17,18].

Primer pairs were designed in these specific regions, when possible, with the Primer3 software [Primer3 Input (version 0.4.0)] and the following criteria: (i) primer length between 18 and 23 nucleotides, (ii) close to the 3′ end when feasible, (iii) melting temperature between 58 and 60 °C when possible, but always avoiding a 2 °C difference between both primers, (iv) product length between 90 and 150 bp, (v) primer GC between 40 and 60% and a GC clamp of 2 and (vi) avoiding primer hairpin, self- and cross-primer dimer formation, checking quality values with the Beacon Designer^™^ program (Premier Biosoft). Primer sequences were also checked for primer specificity using the Primer-Basic Local Alignment Search Tool (blast) against the *Sus scrofa* organism sequence (taxid: 9823) of the RefSeq representative genome database. All designed primers and their features are summarized in Table S2, available in the online Supplementary Material. All oligonucleotides were supplied by Isogen Life Science B.V. (De Meern, The Netherlands).

### cDNA synthesis and Biomark^™^ X9 microfluidic qPCR

Extracted nucleic acids were diluted at specific concentrations for further analysis. Total DNA per sample was prepared at a concentration of 10 ng µl^−1^, while total RNA samples were set at 100 or 50 ng µl^−1^, when possible, and cleansed with the TURBO DNA-*free*^™^ kit (Invitrogen by Thermo Fisher Scientific, Massachusetts, USA). Samples at 100 ng µl^−1^ were included as individual samples, while samples at 50 ng µl^−1^ were pooled per treatment group and sampling time using an equimolar approach to ensure equal representation of each animal. All cleansed RNA individual samples and pools were reverse transcribed using 800 and 650 ng, respectively, into cDNA using the PrimeScript^™^ RT reagent kit in a final volume of 20 µl and following the manufacturer’s protocol (Perfect Real Time, Takara, Japan). A control excluding the reverse transcriptase in the reaction (-RT) was also included to assess non-specific amplification or presence of genomic DNA. Parallelly, DNA samples were also pooled at 10 ng µl^−1^ per treatment group and sampling time for next steps. For DNA samples, pools typically consisted of five animals, while for RNA samples, pools consisted of three animals, subject to the availability of samples meeting the required concentration and quality thresholds. For DNA, available pools covered three sampling times (ST2, ST3 and ST4) per treatment group (G1–G6 and GG) after treatment, while for RNA, pools included two sampling times (ST2 and ST4) per treatment group (G1–G6, GG). A single pool of samples before weaning before treatment (ST1) was also added to the analysis for both DNA and RNA samples, including 5 and 4 animals, respectively, and having a total of 22 and 15 pools, respectively. Besides, an extra pool of DNA samples, including the minimum number of samples to detect the maximum number of genes of the ARG catalogue, was generated to be implemented as a standard (STD), also at 10 ng µl^−1^. Three individual samples with available DNA and RNA extracted material, and ARG abundance and expression data from metagenomics and metatranscriptomics, respectively, were included for comparison analyses (S1, S2 and S3).

Extracted DNA and RNA of individual samples and pools were used to verify and quantify the ARG catalogue presence and expression, respectively, using the microfluidic chip Biomark^™^ X9 System for high-throughput genomics (Standard BioTools, CA, USA). The system employed was the 96.96 IFC chip that allows for 96 individual primer targets and 96 individual samples (48 samples when performing duplicates) to be assessed in a single experiment, using a qPCR technique to amplify target genes of the ARG catalogue from the selected samples. Following the manufacturer’s recommendations, this system includes an initial pre-amplification step of the samples, a treatment to remove components from the previous step and the final quantification of the PCR reactions performed in the IFC. First, DNA and RNA individual samples and pools were pre-amplified using 2 µl of sample and 3 µl of the preamplification pre-mix, including the 96 pairs of primers at 100 µM and the PreAmp Master Mix prepared as in the manufacturer’s protocol (Standard BioTools, South San Francisco, USA). This pre-amplification included an initial activation step of the polymerase for 2 min at 95 °C, followed by a denaturation step for 15 s at 95 °C and an annealing and extension for 4 min at 60 °C. Pre-amplification was performed for 14 cycles for DNA samples and 20 cycles for RNA samples. Second, pre-amplified products were treated with Exonuclease I (New England Biolabs, MA, USA) for 30 min at 37 °C to digest residual primers from the previous step, followed by an inactivation step of the enzyme for 15 min at 80 °C. DNA pre-amplified samples after treatment were diluted 1/20 [adding 93 µl DNA Suspension Buffer (SB, Sigma-Aldrich, MO, USA)/well], while RNA samples were diluted ½ (adding 3 µl DNA SB/well). The pre-amplified STD was diluted 1/5 (adding 18 µl DNA SB) and followed by four ¼ dilutions and two ½ dilutions. Finally, diluted pre-amplified samples were mixed with 2X SsoFast EvaGreen Supermix with Low ROX (Bio-Rad, CA, USA) and 20X Binding Dye to obtain the sample mixes. In parallel, the assay mixes were prepared in a 96-well plate, including 1 µl of each primer pair per well at 100 µM, 2X Loading Reagent (Standard BioTools, South San Francisco, USA) and DNA SB, in a final volume of 40 µl, following the manufacturer’s protocol.

IFC was prepared by injecting the control line fluid as recommended by the protocol, and 5 µl of sample and assay mixes were pipetted into the respective sample and assay inlets on the IFC. The program run in the microfluidic chip Biomark^™^ X9 System was the Gene Expression EvaGreen preset script. Each pool and STD dilutions were assayed in duplicates, and no-template controls (NTC, -RT and water) were included to check for non-specific amplification.

### Relative quantification and data analysis

Amplification data were obtained from the Standard BioTools Real-Time PCR Analysis software v1.0.2 (Standard BioTools, South San Francisco, USA). A quality threshold cut-off value was set at 0.65, and amplification specificities were assessed individually by melting temperature (Tm) analyses per reaction. The Data Analysis Gene (DAG) Expression software v1.0.5.6 [19] was implemented to create standard curves from STD dilutions, obtain *R*-squared values, calculate specific PCR efficiencies and select the most stable genes based on M-value [20]. Since no reference or endogenous genes have been described so far for multi-prokaryotic species samples, normalization was performed for DNA and RNA pool samples separately as follows: (i) considering the same initial nucleic acid concentration, (ii) extracting raw quantity values from the DAG Expression software corrected by PCR efficiencies for each target gene, (iii) measuring M-values for the maximum number of possible amplified genes, (iv) selecting those most stable genes (lowest M-value) and defining a threshold value, (v) calculating the mean quantity values per pool of the most stable genes (normalization mean) and the standard deviation of those means between all pools, (vi) dividing each raw quantity value per gene and pool sample by the selected normalization mean and (vii) showing normalized quantities as log_10_-transformed for proper visualization and comparison between treatment groups and sampling times for the different antibiotic classes. To assess whether normalized ARG quantities per treatment group were significantly higher than the mean of normalized ARG quantities of the other treatment groups, left-sided one-tailed Student’s t-tests were performed for each ARG per treatment group at each sampling time, and for the ST1 pool against all the rest of the pools [21]. To account for the multiple ARG targets and treatment groups, *P*-values were adjusted using the Benjamini–Hochberg procedure for controlling false discovery rate (FDR), and statistical significance was defined as an adjusted *P*-value<0.01 [22].

For individual samples S1, S2 and S3, Spearman’s rank correlation coefficient was used to assess the relationship between the abundance and expression values obtained by qPCR per individual sample, and between these values and the abundance and expression values calculated from the metagenomics and metatranscriptomics data in previous studies [15,16]. The correlation coefficients and the associated *P*-values were computed in R version 4.3.2 using the ‘*cor.test()*’ function that accounts for ties and ensures statistical reliability of the results [23].

## Results

### A catalogue of ARGs defining the piglets’ gut resistome

**Fig. 2.**
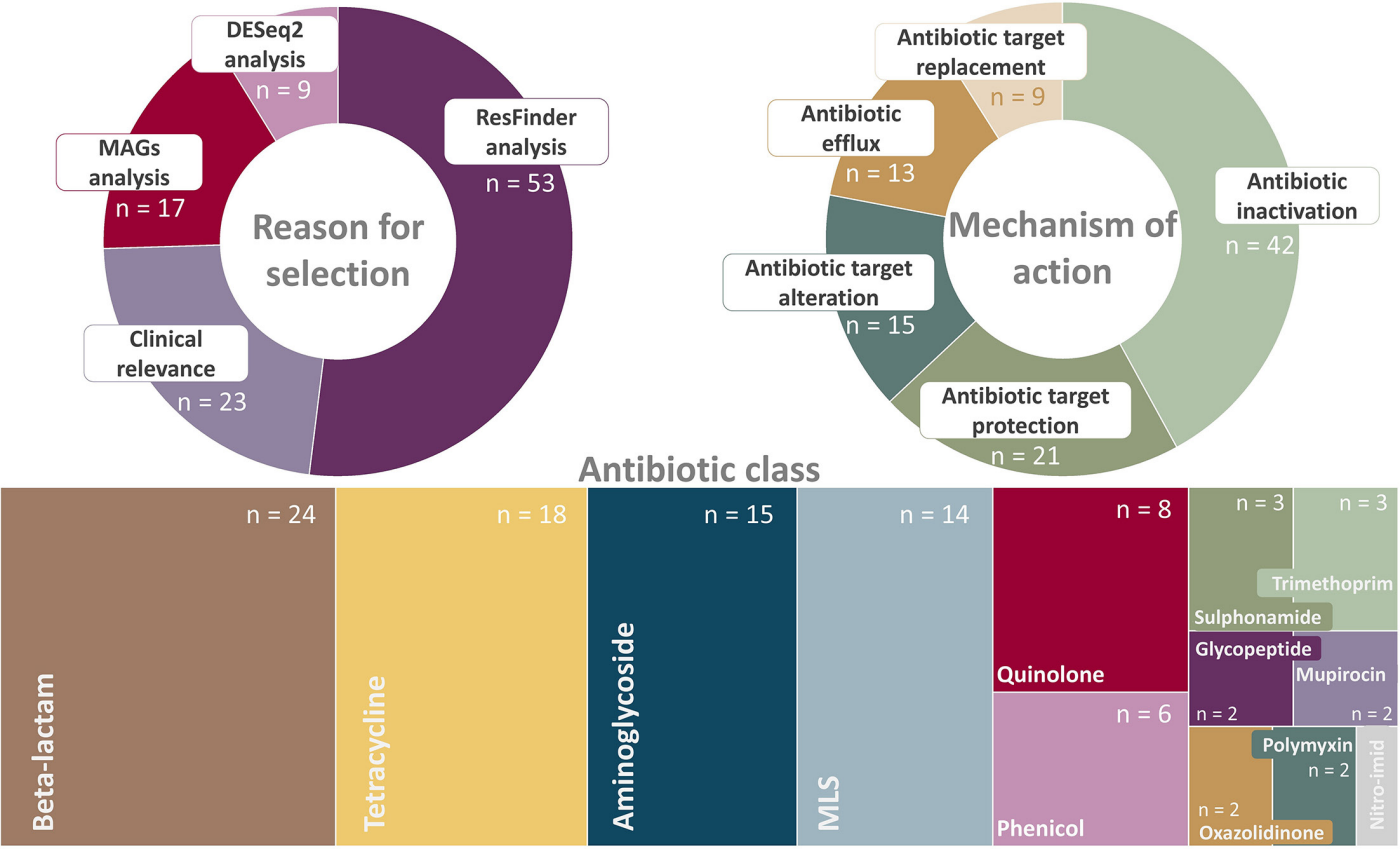
Overview of the ARG catalogue, including 102 genes classified by reason for selection, antibiotic class and mechanism of action.

The ARG catalogue generated in this study comprehended a total of 102 genes that could be detected using a set of 93 primers (Table S2). From these, 53 ARGs were selected from the metagenomics data identified in at least 20% of the samples (*n*≥56) [15], 17 ARGs were selected from their direct identification in the MAGs, and 9 ARGs were found to be differentially expressed between treatment groups from the DESeq2 analyses [16]. The remaining ARGs (*n*=23) were selected for conferring resistance to clinically relevant antibiotic classes. The catalogue integrated genes conferring resistance to most antibiotic classes, including beta-lactams (*n*=24), tetracyclines (*n*=18), aminoglycosides (*n*=15), macrolides-lincosamides-streptogramins (MLS, *n*=14), quinolones (*n*=8) and phenicol (*n*=6), among others. A schematic overview of the features of the genes included in the ARG catalogue is represented in Fig. 2, and specific information is detailed in Table S2.

Multiple gene alignments allowed the design of primers in conserved regions to cover several ARGs with a single pair of primers. For example, one pair of primers could detect *cfxA* and *cfxA3* genes, whereas another pair covered *tet*(O) and *tet*(O/32/O) genes. In the case of beta-lactamase-encoding genes, alignments of all variants were considered to design primers in highly conserved regions to capture most of them. For example, out of the 85 available *bla*_VIM_ gene sequences in CARD, all genes except for *bla*_VIM-7_, *bla*_VIM-61_ and *bla*_VIM-69_ could be detected with the designed primers. Three endogenous genes were also included (V7_16S rRNA, *gyrA* and *rpoD*) as positive controls. Despite *gyrA* and *rpoD* having been widely implemented as endogenous reference genes [24,25], gene alignments of multi-prokaryotic species did not identify conserved regions to design primers from metagenomic samples.

### Primer specificity and PCR efficiency

The STD pool allowed for the generation of 93 standard curves out of the total 96 primer pairs. The comparison of the Ct values of these 93 standard curves revealed high variability between genes (Fig. S1) and identified 13 genes for which amplification was not optimal (see below). The remaining 80 calibration curves produced linear standard curves, with high coefficients of determination (*R^2^* >0.95), except for two genes: *dfrA20* (*R^2^=*0.8957) and *vanA* (*R^2^=*0.8485). Considering the slopes of the resulting trend lines, PCR amplification efficiencies for the 80 genes ranged from 73 to 110%. Melting curve analyses confirmed a single specific amplicon for most amplified gene products, although multiple peaks were observed in primers designed to identify multiple genes. For 27 genes, amplifications in the control excluding the reverse transcriptase (-RT) samples occurred and were further assessed. In most cases where amplification was observed in -RT controls, it occurred significantly later than in the experimental samples. Specifically, while the most diluted STD had a Ct below 14, the -RT samples amplified an average of 10.4 Cts later, with differences ranging from 6.2 to 14.6 cycles. These late amplifications indicate that any residual genomic DNA or non-specific products were present at low levels and did not affect the amplification of experimental samples. Regarding the 3 genes that did not generate a standard curve (*bla*_OXA_, *bla*_GES_ and *mecC/PBP2C*) and the 13 genes that did not amplify properly [*bla*_CMY_, *bla*_IMP_, *bla*_KPC_, *bla*_NDM_, *bla_VIM_*, *cfrA*, *mcr-4*, *mepA*, *mph*(N), *mupA*, *mupB*, *qnrA* and *tet*(45)], half of these genes were selected for being clinically relevant and were not present in our samples. The other half were selected from the MAGs or the DESeq2 analyses. For six of these genes, qPCRs performed in 96-well plates with positive controls showed high coefficients of determination and PCR amplification efficiencies: *bla*_OXA_ (*R^2^*=0.9995, 94.3%), *bla*_GES_ (*R^2^*=0.9939, 89.4%), *bla*_CMY_ (*R^2^*=0.9971, 108.9%), *bla*_KPC_ (*R^2^*=0.9998, 95.0%), *bla*_NDM_ (*R^2^*=0.9993, 96.2%) and *mcr-4* (*R^2^*=0.9970, 94.5%).

### Correlation of abundance and expression profiles of individual samples

**Fig. 3.**
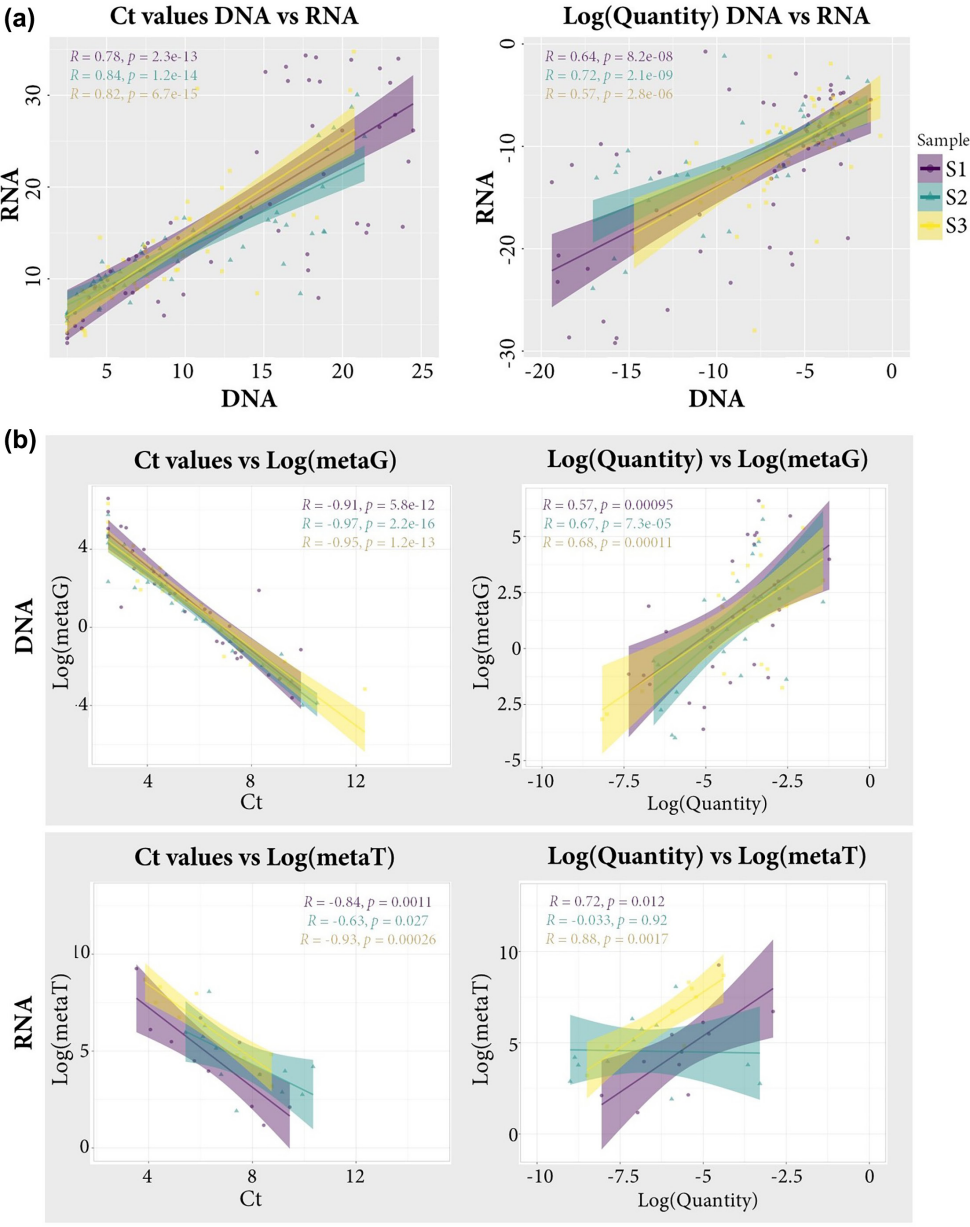
(a) Correlation analysis between the DNA and RNA extracted material of the same three individual samples (**S1, S2 and S3**) comparing the Ct values and the log_10_-transformed quantity values. (**b) **Correlation analyses between the metagenomics prevalence data and the Ct and log_10_-transformed quantity values of three DNA samples, and between the metatranscriptomics expression data and the Ct and log_10_-transformed quantity values of three RNA samples. Sequencing prevalence and expression data were obtained from ResFinder software and calculated and normalized as described in Guitart-Matas *et al*. [15,16]. Correlation is measured using Spearman’s rank method that accounts for ties in the data.

Three individual samples (S1, S2 and S3) with available DNA and RNA extracted material and shotgun metagenomics and metatranscriptomics data were included in the microfluidic qPCR for comparison analyses. First, the correlation between the Ct values and the quantity values obtained for the DNA and RNA extracted material of the same individual sample were compared. A significant and strong correlation (0.84>*R*>0.78, *P-adj* <0.01) was observed between the Ct values obtained by qPCR for the DNA and RNA of individual samples, respectively. This correlation remained positive when comparing log_10_-transformed quantity values (0.72>*R*>0.57, *P-adj <0.01*) (Fig. 3a). These values were also compared against the log_10_-transformed values of the metagenomics data (metaG) for DNA-extracted samples and the log_10_-transformed values of the metatranscriptomics data (metaT) for RNA-extracted samples calculated from ResFinder as described in previous studies [15,16]. From the 53 ARGs included in the catalogue from the ResFinder analysis, a total of 33 different ARGs could be compared in this analysis for DNA samples and 17 for RNA samples. Again, a stronger significant correlation was observed when comparing Ct values to the sequencing data than when comparing log_10_-transformed quantity values. A strong negative correlation was observed between the log_10_-transformed metagenomic values and the Ct values of DNA samples with *R* coefficient values ranging from −0.91 to −0.97 (*P-adj* <0.01) (Fig. 3b). For the RNA samples, this strong negative correlation was significant in two of the samples with −0.84 and −0.93 coefficient values (*P-adj*=0.0011 and 0.0003, respectively). These two samples also showed high positive correlation when comparing the log_10_-transformed quantity values and the log_10_-transformed metatranscriptomic expression levels measured with ResFinder, with *R* coefficient values of 0.72 (*P-adj*=0.012) and 0.88 (*P-adj*=0.0017), ensuring the validity of the developed qPCR microfluidic panel (Fig. 3b).

### Gene stability evaluation for normalization of gene amplification data

Since no endogenous genes with consensus DNA sequence have been described for samples with multi-prokaryotic species, normalization of gene amplification data could not be performed assuming constant expression of a single gene across samples. In our study, the 16S ribosomal RNA gene showed excessively low Ct values for all the samples, averaging 2.5, which represents the lower detection limit of the system. This indicated an overabundance of this target gene exceeding the optimal linear range and consequently discarding it as a candidate for normalization of the amplification data. Therefore, normalization of the gene amplification data was performed based on gene stability, or M-values, across samples. The means of relative amounts of the most stable genes, defined here as the normalization means, were used to normalize the relative amount of target genes in each sample. To ensure the reliability of this approach, normalization means were calculated using the top 10, 20, 30 and 40 most stable genes for DNA pool samples. While the absolute average values varied, the resulting data matrices showed high consistency, indicating that the normalization was not sensitive to the number of stable genes used. For RNA pool samples, normalization mean could be calculated for a maximum of 30 genes.

Here, the 20 most stable genes for both DNA and RNA pool sample sets were chosen for normalization, as the lowest standard deviation of normalization means between pools was observed for the DNA top 20 (sd=0.0210). M-values of the 20 most stable genes for both DNA and RNA pool samples are listed in Table 1.

**Table 1.** M-values of the 20 most stable genes of the DNA and RNA pool sample sets. Bold genes indicate that they are common in both

Most stable genes in DNA pool samples		Most stable genes in RNA pool samples	
**Gene**	**M-value**	**Gene**	**M-value**
***tet*(W/32/O)**	0.6398	***tet*(40)**	1.2777
***tet*(40)**	0.6436	***tet*(O/W)**	1.2849
*tet*(O)/*tet*(O/32/O)	0.6585	*aph(3')-Ia*	1.2897
***tet*(O/W)**	0.6634	***aph(3')-III***	1.3244
***aph(3')-III***	0.6700	***tet*(44)**	1.3468
***tet*(W)**	0.6709	***cfxA/cfxA3***	1.3889
***tet*(Q)**	0.7478	***tet*(Q)**	1.3929
*ant(6)-Ib*	0.7493	***tet*(W)**	1.4081
***tet*(44)**	0.7569	***cfr*(E)**	1.4286
***cfxA/cfxA3***	0.7789	*catA1*	1.4921
*bla*_ACI-1_	0.8232	***erm*(B)**	1.5671
***cfr*(E)**	0.8395	*cfxA6*	1.6264
***erm*(F)**	0.8964	*tet*(C)	1.753
***erm*(B)**	0.9391	***erm*(F)**	1.9068
*erm*(G)	0.9767	*nimJ*	1.9112
*tetA*(P)	1.0445	*fexA*	1.9755
***tetB*(P)**	1.063	***cfr*(C)**	2.0191
***cfr*(C)**	1.0697	***tet*(W/32/O)**	2.047
***tet*(X)**	1.0841	***tet*(X)**	2.0882
*rmtD2*	1.1961	***tetB*(P)**	2.2065

### Gene abundance analyses of DNA pool samples by microfluidic qPCR

**Fig. 4.**
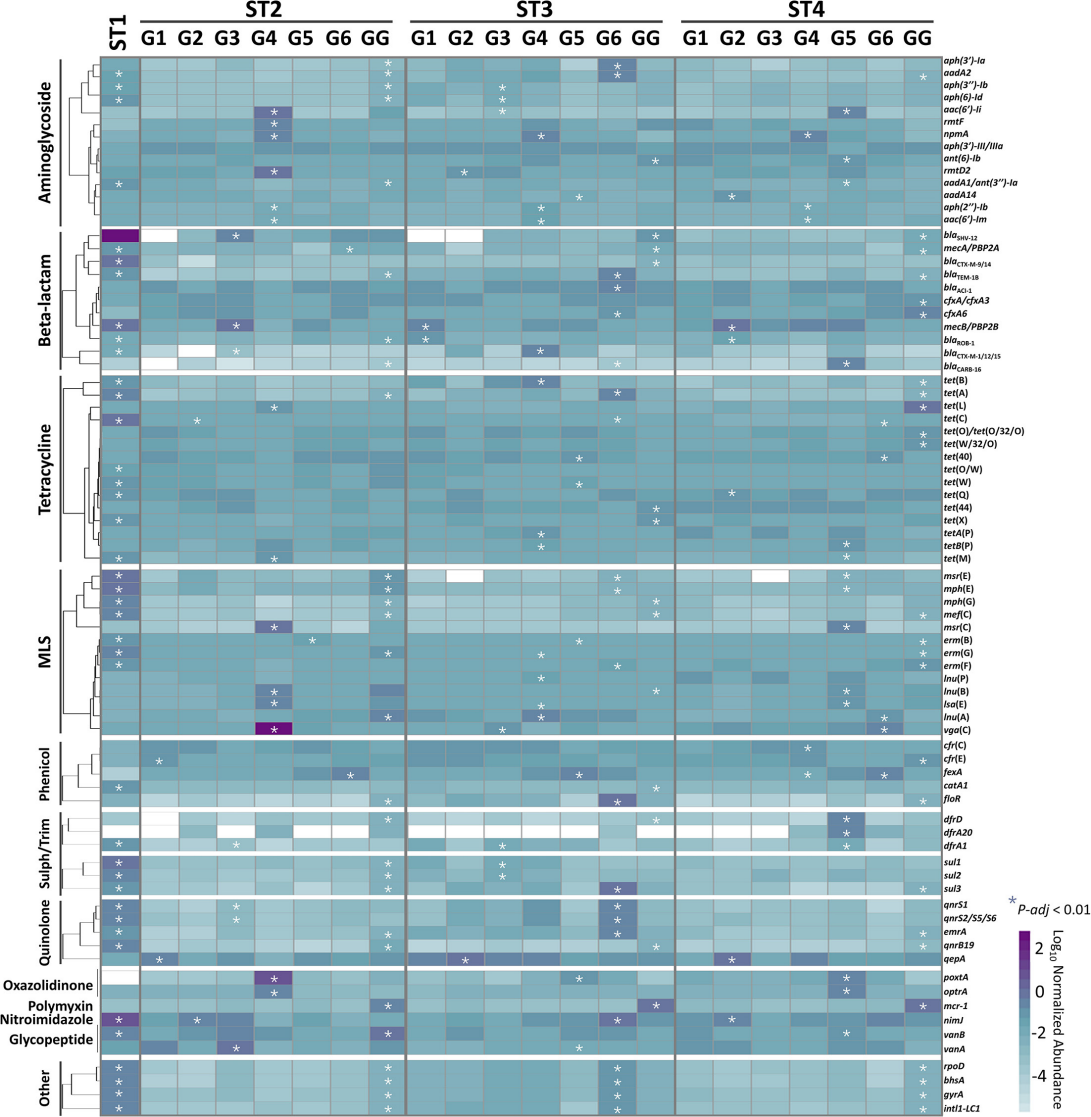
Heatmap representing normalized relative abundance of ARGs by microfluidic qPCR per treatment group (**G1–G6, GG**) and sampling time (ST1–ST4). A unique pool for ST1 was analysed. The relative standard curve method was applied for relative quantification using the normalization mean of the 20 most stable genes. ARGs were grouped by antibiotic class. MLS corresponds to the macrolide-lincosamide-streptogramin group of antibiotics. White asterisks indicate significant differences between individual treatment groups and the mean of all remaining groups at each sampling time (adjusted *P*<0.01). For ST2, ST3 and ST4, comparisons were performed within each sampling time, whereas ST1 was compared against the global mean of the remaining pools. All *P*-values were corrected for multiple testing across all ARGs and treatment groups using the Benjamini–Hochberg procedure. G1, trimethoprim/sulfamethoxazole; G2, colistin; G3, oral vaccination; G4, gentamicin; G5, untreated control with water acidification; G6, untreated control; GG, amoxicillin (farm of origin); ST1, 1 day before weaning; ST2, 3 days post-treatment; ST3, 2 weeks post-treatment; ST4, 4 weeks post-treatment.

ARG abundance profiles of DNA pool samples were compared between treatment groups per sampling time for each resistance gene. Excluding the 16S rRNA gene, relative abundances of the 79 different genes grouped by antibiotic class are shown in Fig. 4, and significant results at a 99% confidence level (*α*=0.01) are represented with a white asterisk after FDR correction. For detailed statistical analysis results, see Table S3. For the ARGs conferring resistance to aminoglycosides, an increase of *aac(6’)-Ii*, *rmtF*, *npmA*, *rmtD2*, *aph(2’’)-Ib* and *aac(6’)-Im* (*P-adj* <0.01) was observed in the gentamicin-treated group (G4) at 3 days post-treatment (ST2) compared to the other treatment groups. At 2 (ST3) and 4 weeks (ST4) post-treatment, only the increase of *npmA*, *aph(2’’)-Ib* and *aac(6’)-Im* was significant (*P-adj* <0.01) in G4 in comparison with the other treatment groups. Also, an increase of the *aph(3’)-Ia* and *aadA2* genes was detected in the untreated control group (G6) at 2 weeks post-treatment (ST3) and the *aac(6’)-Ii*, *ant(6)-Ib* and *aadA1/ant(3’’)-Ia* genes in the control group with water acidification (G5) at 4 weeks post-treatment (ST4) when comparing to the rest of treatment groups at each sampling time (*P-adj* <0.01) (Fig. 4). For the ARGs conferring resistance to beta-lactams, *bla*_SHV-12_, *mecA/PBP2A*, *bla*_CTX-M-9/14_, *bla*_TEM-1B_, *mecB/PBP2B*, *bla*_ROB-1_ and *bla*_CTX-M-1/12/15_ were found to be more abundant in the pooled sample at pre-weaning before treatment (ST1) compared to ST2, ST3 and ST4 (*P-adj* <0.01). After treatment, the higher abundance of the methicillin resistance *mecB/PBP2B* gene was detected in the vaccinated group (G3) 3 days post-treatment (ST2), in the trimethoprim/sulfamethoxazole-treated group (G1) at 2 weeks post-treatment (ST3) and in the colistin-treated group (G2) 4 weeks post-treatment (ST4) (*P-adj* <0.01). An increase of other beta-lactamases was also observed throughout the experiment in several treatment groups. Genes conferring resistance to cefotaxime (*bla*_CTX-M-1/12/15_) and carboxypenicillin beta-lactams (*bla*_CARB-16_) were detected to be higher in the gentamicin-treated group (G4) 2 weeks post-treatment (ST3) and the control group with water acidification (G5) at 4 weeks post-treatment (ST4), respectively (*P-adj* <0.01). At 2 weeks post-treatment (ST3), a higher abundance of the genes encoding for beta-lactamases *bla*_TEM-1B_, *bla*_ACI-1_ and *cfxA6* was observed in the untreated control group (G6). Also, higher prevalence (*P-adj* <0.01) of *bla*_SHV-12_, *mecA/PBP2A*, *bla*_TEM-1B_, *cfxA/cfxA3* and *cfxA6* was also detected in the group that remained at the farm of origin treated with amoxicillin (GG) at 4 weeks post-treatment (ST4). Regarding the ARGs conferring resistance to tetracyclines, fewer differences were observed between treatment groups and sampling times. However, higher abundances of *tet*(B), *tet*(A), *tet*(C), *tet*(O/W), *tet*(W), *tet*(Q) and *tet*(X) were observed before weaning (ST1) (*P-adj* <0.01). Other genes conferring resistance to tetracyclines were also higher at ST2, ST3 and ST4 when comparing treatment groups. For example, a higher amount of *tet*(B), *tetA*(P) and *tetB*(P) and *tet*(A) and *tet*(C) was detected in the gentamicin-treated group (G4) and the untreated control group (G6), respectively, 2 weeks post-treatment (ST3). By the end of the experiment, at 4 weeks post-treatment (ST4), most significant differences (*P-adj* <0.01) were observed in the group that remained at the farm of origin treated with amoxicillin (GG), showing a higher amount of *tet*(B), *tet*(A), *tet*(L), *tet*(O)/*tet*(O/32/O) and *tet*(W/32/O) compared to the other treatment groups (G1–G6) at 4 weeks post-treatment (ST4) (Fig. 4).

For the rest of ARGs analysed here, a higher abundance of ARGs conferring resistance to MLS [*msr*(E), *mph*(E), *mph*(G), *mef*(C), *erm*(B), *erm*(G) and *erm*(F)], sulphonamides (*sul1*, *sul2* and *sul3*), quinolones (*qnrS1*, *qnrS2/S5/S6*, *emrA* and *qnrB19*), nitroimidazole (*nimJ*) and vancomycin (*vanB*) was observed before weaning (ST1) when comparing to ST2, ST3 and ST4 (*P-adj* <0.01). Moreover, four ARGs conferring resistance to MLS [*msr*(C), *lnu*(B), *lsa*(E) and *vga*(C)] and two oxazolidinone resistance genes (*optrA* and *poxtA*) were found to be in a higher amount in the gentamicin-treated group (G4) at 3 days post-treatment (ST2). At 2 weeks (ST3) and 4 weeks (ST4) post-treatment, the *qepA* gene, conferring resistance to quinolones, was found to be increased in the colistin-treated group (G2) (*P-adj* <0.01). Also, multiple genes conferring resistance to MLS, trimethoprim, oxazolidinones and vancomycin were observed to be higher (*P-adj* <0.01) in the control group with water acidification (G5) 4 weeks post-treatment (ST4). Besides, 2 weeks post-treatment (ST3), the untreated control group (G6) showed higher abundance (*P-adj* <0.01) of the chloramphenicol resistance gene *floR*, the sulphonamide resistance gene *sul3*, the nitroimidazole resistance gene *nimJ* and three quinolone resistance genes (*qnrS1*, *qnrS2/S5/S6* and *emrA*). Finally, the group that remained at the farm of origin treated with amoxicillin (GG) showed higher abundance (*P-adj* <0.01) of genes conferring resistance to MLS, chloramphenicol, trimethoprim, sulphonamide, quinolone and colistin at multiple sampling times (ST2, ST3 and ST4). The presence of the colistin resistance gene *mcr-1* was higher (*P-adj* <0.01) at all sampling times (ST2, ST3 and ST4) for the group that remained at the farm of origin treated with amoxicillin (GG) when compared to the rest of treatment groups (G1–G6) (Fig. 4).

### Gene expression analyses of RNA pool samples by microfluidic qPCR

**Fig. 5.**
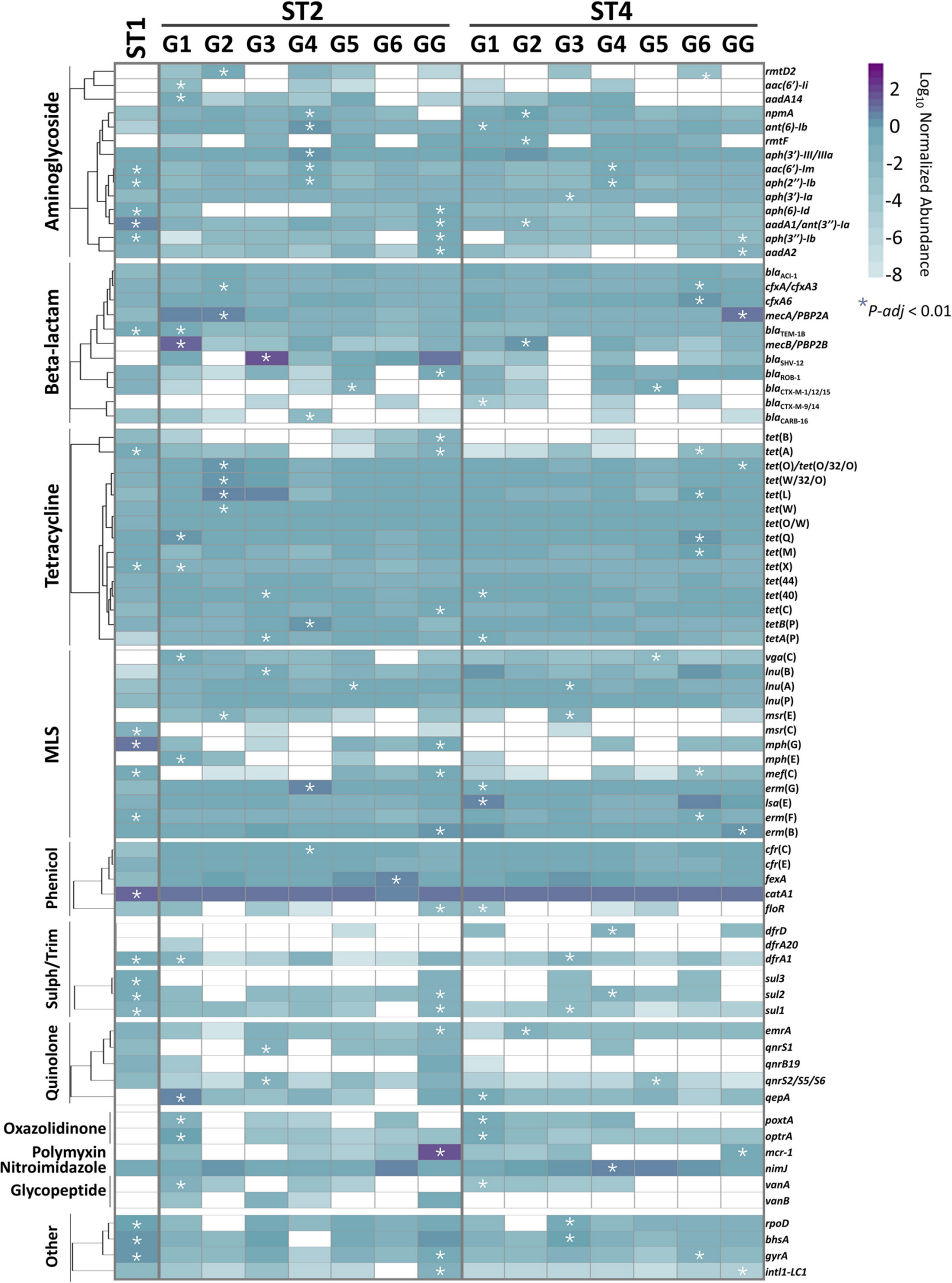
Heatmap representing normalized gene expression of ARGs by microfluidic qPCR per treatment group (**G1–G6, GG**) and sampling time (ST1, ST2 and ST4). A unique pool for ST1 was analysed. The relative standard curve method was applied for relative expression quantification using the normalization mean of the 20 most stable genes. ARGs were grouped by antibiotic class. MLS corresponds to the macrolide-lincosamide-streptogramin group of antibiotics. White asterisks indicate significant differences between individual treatment groups and the mean of all remaining groups per sampling time (adjusted *P*<0.01). For ST2, ST3 and ST4, comparisons were performed within each sampling time, whereas ST1 was compared against the global mean of the remaining pools. All *P*-values were corrected for multiple testing across all ARGs and treatment groups using the Benjamini–Hochberg procedure. G1, trimethoprim/sulfamethoxazole; G2, colistin; G3, oral vaccination; G4, gentamicin; G5, untreated control with water acidification; G6, untreated control; GG, amoxicillin (farm of origin). ST1, 1 day before weaning; ST2, 3 days post-treatment; ST3, 2 weeks post-treatment; ST4, 4 weeks post-treatment.

Expression of ARG profiles of RNA pool samples was compared between treatment groups per sampling time for each resistance gene. Relative gene expression of the 79 different genes grouped by antibiotic class is shown in Fig. 5, and significant results at a 99% confidence level (*α*=0.01) are represented with a white asterisk after FDR correction. For detailed statistical analyses results, see Table S4. At the pre-weaning stage (ST1), higher expression levels of at least one gene conferring resistance to aminoglycosides, beta-lactams, tetracyclines, MLS, phenicol, trimethoprim and sulphonamides were identified when compared with the rest of sampling times (*P-adj*<0.01). Three days post-treatment (ST2), in the gentamicin-treated group (G4), higher expression levels of the aminoglycoside resistance genes, including *ant(6)-Ib*, *rmtF*, *aph(3’)-III/IIIa*, *aac(6’)-Im* and *aph(2’’)-Ib*, were detected when compared to other treatment groups (*P-adj*<0.01). At this sampling time (ST2), the group treated with amoxicillin (GG) and remaining at the farm of origin showed higher expression levels of other aminoglycoside resistance genes such as *aph(6)-Id*, *aadA1/ant(3’’)-Ia*, *aph(3’’)-Ib* and *aadA2* (*P-adj*<0.01). For the beta-lactam resistance genes, higher expression levels of the methicillin resistance genes *mecA/PBP2A* and *mecB/PBP2B* were observed in the colistin-treated (G2) and trimethoprim/sulfamethoxazole-treated (G1) groups 3 days post-treatment (ST2), respectively (*P-adj*<0.01). The *mecA/PBP2A* gene was also more expressed in the group that remained at the farm of origin treated with amoxicillin (GG) 4 weeks post-treatment (ST4). Moreover, the beta-lactam resistance gene *bla*_SHV-12_ was found to be more expressed in the vaccinated group (G3) 3 days post-treatment (ST2) in comparison to the other treatment groups (*P-adj*<0.01). Besides, 3 days post-treatment (ST2), the tetracycline resistance genes *tet*(O)/*tet*(O/32/O), *tet*(W/32/O), *tet*(L) and *tet*(W) showed higher expression in the colistin-treated group (G2), and the *tet*(Q) and *tet*(X) genes in the trimethoprim/sulfamethoxazole-treated group (G1) (*P-adj*<0.01). Also, the untreated control group (G6) showed higher expression of the *tet*(A), *tet*(L), *tet*(Q) and *tet*(M) genes 4 weeks post-treatment (ST4) (*P-adj*<0.01) (Fig. 5).

For the MLS antibiotic class, the gentamicin-treated group (G4) showed a higher expression of the resistance gene *erm*(G) 3 days post-treatment (ST2), while the trimethoprim/sulfamethoxazole-treated group (G1) showed higher expression of the *erm*(G) and *lsa*(E) genes at 4 weeks post-treatment (ST4) (*P-adj*<0.01). Moreover, the *erm*(B) gene was more highly expressed in the group that remained at the farm of origin treated with amoxicillin (GG) at 3 days (ST2) and 4 weeks post-treatment (ST4) in comparison to the other treatment groups (*P-adj*<0.01). The *catA1* gene, conferring resistance to chloramphenicol, was expressed in all treatment groups at all sampling times. Regarding trimethoprim, sulphonamides and quinolones, several genes were not expressed in all treatment groups, represented in blank. However, the group that remained at the farm of origin treated with amoxicillin (GG) showed expression of all tested sulphonamide and quinolone resistance genes, with a higher expression of *sul1*, *sul2* and *emrA* genes at 3 days post-treatment (ST2) compared to the other treatment groups (*P-adj*<0.01). Moreover, *mcr-1* was more expressed (*P-adj*<0.01) at this timepoint and at 4 weeks post-treatment (ST4) in comparison to the other treatment groups. The nitroimidazole resistance gene *nimJ* was also expressed in all treatment groups at all sampling times but was found to be higher (*P-adj*<0.01) in the gentamicin-treated group (G4) at 4 weeks post-treatment (ST4) (Fig. 5).

## Discussion

Reducing antimicrobial use in farms is crucial to preserve the action of current antimicrobials and mitigate the emergence of AMR. Achieving this goal requires a comprehensive and multi-faceted approach, from improving husbandry practices and biosecurity to optimizing disease control and prevention strategies, investigating alternative therapies and strengthening monitoring and surveillance. The implementation of these strategies collectively would help minimize reliance on antimicrobials in farms, ensuring the sustainability of livestock production while promoting animal welfare [26,28]. Within this context, the study performed herein aimed to develop a high-throughput screening tool for AMR surveillance in pig faecal samples, by implementing a microfluidic platform and assessing the associated challenges and constraints. The development and validation of this tool have been derived from results obtained in previously published metagenomics and metatranscriptomics analyses [15]. These two studies allowed the generation of an ARG catalogue containing more than 100 ARGs and comprising the pig resistome. In our approach, ResFinder v4.2.5 software and database were used to identify specific variants of ARGs and calculate read and transcript abundances as previously described [15,29]. Moreover, this strategy allowed performing gene alignments between related ARGs of the pig resistome and further optimizing primer design for multiple genes. Similarly, attempts were made to identify conserved regions in endogenous genes (*gyrA*, *rpoD*) across multiple species for normalization purposes, though conserved regions were not identified. Consequently, these genes were also not among the most stable for normalization. Besides, the 16S rRNA gene could not be used for normalization in our study since Ct values were outside the detecting threshold. Therefore, gene stability values (M-value) were assessed to determine their suitability for standardization. Genes with the lowest average expression stability within our dataset were ARGs mainly conferring resistance to tetracyclines, aminoglycosides and macrolides. These ARGs were detected in all pools following a similar expression pattern, and some, such as *tet*(W), *tet*(Q), *tet*(44), *tet*(40) and *erm*(B), have been previously reported in high abundance in other swine studies [30,31]. Overall, the mean normalization method carefully evaluated and conducted in this study ensured the generation of reliable results that allowed for a biological interpretation of the data.

Another key challenge of the methodology performed herein was determining the optimal initial concentration and consequent dilution factors to accurately detect the ARGs in both DNA and RNA samples within the standard curves of each target gene. From the total 96 primer pairs included in the microfluidic qPCR chip, 80 showed proper amplification of the standard curves with optimal primer amplification within our samples. As stated before, apart from the V7_16S rRNA gene that was overly concentrated in all DNA and RNA samples leading to a saturated amplification curve, the rest of the genes could be accurately detected within the specific range. Therefore, the implemented initial concentrations and dilutions for DNA and RNA individual and pool samples, as well as the number of pre-amplification step cycles performed, were appropriate for our type of samples, containing multiple species and high microbial diversity. The use of relative standard curves enabled us to compare ARG abundances and expression levels between treatment groups and sampling times per each ARG.

Further evidence supporting the reliability of the qPCR array developed in this study was the strong correlation observed between the abundance and expression levels of ARGs detected using the microfluidic platform and those identified through metagenomics and metatranscriptomics sequencing in our previous research [15]. Based on the available literature, no prior studies have conducted correlation analyses comparing these methodologies for detecting and quantifying ARGs. Different observations from these analyses can be highlighted. First, as expected, a significant and strong positive correlation is observed between the DNA and the RNA of the same animal at the same sampling time (0.78–0.84). Second, a strong negative correlation (0.91–0.97) was observed between the Ct values and the log_10_-transformed metagenomics sequencing data. The same pattern was observed for both DNA and RNA Ct values compared to the metagenomics and metatranscriptomics data, respectively. However, due to the lower number of genes expressed in the RNA samples and/or detected in the metatranscriptomics dataset, the test was found to be less statistically significant in this case (0.63–0.93). Third, using quantity values (relative to the standard curves) to perform these correlations resulted in a weaker association between these values and the log_10_-transformed sequencing data (0.57–0.88). This last observation could be explained by the fact that the standard curves performed here and used to extrapolate values were generated from a pool with multiple samples. Future studies could consider the implementation of synthetic oligonucleotide sequences for each gene with equal amplicon concentrations, which would also allow abundance and expression level comparisons between ARGs.

Therefore, these findings support previous bioinformatic pipelines and the microfluidic platform designed herein for the detection and quantification of ARGs, highlighting their potential as complementary tools for more comprehensive analyses. Notably, our microfluidic qPCR approach consistently detected nearly all assessed ARGs, including several targets that were not captured by shotgun sequencing in specific samples. This suggests that the platform provides a lower limit of detection for less abundant genes compared to shotgun sequencing at standard depths. Alternatively, increasing sequencing depth could help validate this assumption, albeit it may be limited by cost-effectiveness concerns. On the other hand, sequencing technologies can provide valuable insight into the genetic context of ARGs, which is crucial for determining whether they are mobile or associated with a specific pathogen, fundamental for comprehensive risk assessment.

In this study, almost all ARGs included in the microfluidic platform were detected in DNA pool samples of all treatment groups and sampling times, albeit not all appeared to be expressed in the RNA pool samples. Moreover, since our samples included multi-prokaryotic species, a higher expression within our pools may either indicate a higher presence of a specific species expressing a particular ARG or higher transcription of those genes with no change in species abundance. Taken together, the impact of gentamicin treatment (G4) on the resistome at 3 days post-treatment (ST2), previously observed in metagenomics and metatranscriptomics data [15], was also confirmed at both genomic and transcriptomic levels in this study. Similar observations occurred for the group treated with amoxicillin that remained at the farm of origin (GG) at 4 weeks post-treatment (ST4). This group showed the highest number of differences at the genomic level with genes conferring resistance to most antibiotic classes tested. Besides, metagenomic sequencing revealed the presence of the *mcr-1* gene, which confers resistance to colistin, in this treatment group (GG) 2 weeks post-treatment (ST3) [15]. Notably, in this study, *mcr-1* was not only detected at the genomic level in this group (GG) but also exhibited a significantly higher expression at the transcriptomic level, indicating active expression at 3 days (ST2) and 4 weeks (ST4) post-treatment. Given that colistin is a last-resort antibiotic for the treatment of multidrug-resistant bacteria, the detection of both the prevalence and expression of this gene under continuous amoxicillin pressure raises a significant concern. This is particularly remarkable considering that no polymyxins were administered to these animals or their sows and that colistin consumption in Spain has decreased nearly 100% since 2016, after the implementation of the REDUCE Spanish programme [32,33]. Since the abundance and expression of this gene at the experimental farm are significantly lower in comparison to the values detected at the farm of origin, its presence may be linked to the conditions in the farm of origin. This hypothesis could be validated through sampling workers, animal water and feed and the husbandry environment, underscoring the relevance of implementing a One Health perspective for proper AMR surveillance [34,35].

Overall, the cost-effective and highly sensitive microfluidic Biomark^™^ X9 System validated in this study enables high-throughput analysis, allowing for simultaneous assessment of multiple ARGs and numerous samples, offering a critical advantage for large-scale surveillance studies. Moreover, since the entire workflow can be adapted to diverse sample types, such as water, manure, or hospital samples, our approach offers broad applicability across different environments, aligning with the intersectoral goals of One Health.

## Supplementary material

10.1099/mgen.0.001755 Supplementary Material 1.

10.1099/mgen.0.001755 Supplementary Material 2.

## Abbreviations

AMR: antimicrobial resistance
ARGs: antimicrobial resistance genes
CARD: Comprehensive Antibiotic Resistance Database
DAG: Data Analysis Gene
FDR: false discovery rate
IRTA: *Institut de Recerca i Tecnologia Agroalimentàries*
MAGs: metagenome-assembled genomes
PWD: post-weaning diarrhoea
STD: standard
