# Supplementary material for: Implementation of a high-throughput microfluidic platform for antimicrobial resistance surveillance in swine production systems
Source: Microb Genom. 2026 Jun 15;12(6):001755. doi: 10.1099/mgen.0.001755 (PMC13268206; doi:10.1099/mgen.0.001755)
Supplement: Supplementary Material 1. [file mgen-12-01755-s001.pdf]

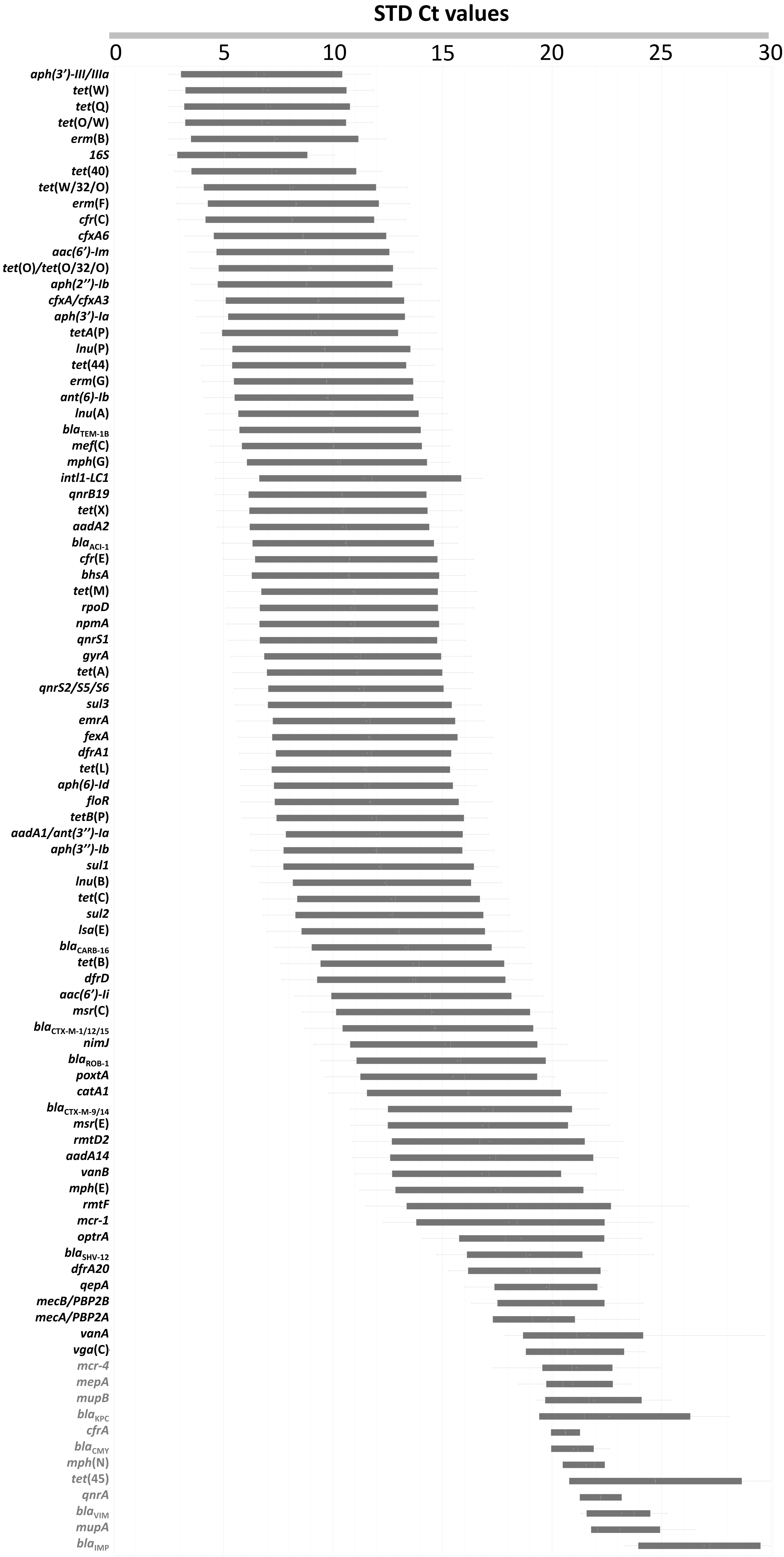

Supplementary Figure S1. Ct values obtained to generate 93 standard (STD) curves for all genes. Genes that did not amplify properly are coloured in grey.
